# Protein Arginine Methyltransferases as Therapeutic Targets in Hematological Malignancies

**DOI:** 10.3390/cancers14215443

**Published:** 2022-11-05

**Authors:** Camille Sauter, John Simonet, Fabien Guidez, Baptiste Dumétier, Baptiste Pernon, Mary Callanan, Jean-Noël Bastie, Romain Aucagne, Laurent Delva

**Affiliations:** 1 Inserm U1231, Team Epi2THM, LipSTIC Labex, UFR des Sciences de Santé, Université de Bourgogne, Université Bourgogne Franche-Comté, 21000 Dijon, France; 2 Unit for Innovation in Genetics and Epigenetic in Oncology (IGEO)/CRIGEN Core Facility, University Hospital François Mitterrand, 21000 Dijon, France; 3 Department of Clinical Hematology, University Hospital François Mitterrand, 21000 Dijon, France

**Keywords:** hematological malignancies, arginine methylation, PRMT, inhibitors, clinical trials

## Abstract

**Simple Summary:**

This review summarizes the current findings about the cellular roles of protein arginine methyltransferases (PRMTs) in the formation of blood cells from stem cell progenitors and in different blood cancers such as leukemia and lymphoma. The work also provides a substantial insight into the development and the use of PRMT inhibitors for the treatment of hematological cancers. This review aims to show the research community that targeting PRMTs could be a novel and promising therapeutic approach for these diseases.

**Abstract:**

Arginine methylation is a common post-translational modification affecting protein activity and the transcription of target genes when methylation occurs on histone tails. There are nine protein arginine methyltransferases (PRMTs) in mammals, divided into subgroups depending on the methylation they form on a molecule of arginine. During the formation and maturation of the different types of blood cells, PRMTs play a central role by controlling cell differentiation at the transcriptional level. PRMT enzymatic activity is necessary for many cellular processes in hematological malignancies, such as the activation of cell cycle and proliferation, inhibition of apoptosis, DNA repair processes, RNA splicing, and transcription by methylating histone tails’ arginine. Chemical tools have been developed to inhibit the activity of PRMTs and have been tested in several models of hematological malignancies, including primary samples from patients, xenografts into immunodeficient mice, mouse models, and human cell lines. They show a significant effect by reducing cell viability and increasing the overall survival of mice. PRMT5 inhibitors have a strong therapeutic potential, as phase I clinical trials in hematological malignancies that use these molecules show promising results, thus, underlining PRMT inhibitors as useful therapeutic tools for cancer treatment in the future.

## 1. Introduction

### 1.1. Arginine Methylation

Arginine methylation has been discovered more than 50 years ago on histone proteins [1] and has become a subject of interest in recent years. The methylation of arginine on histone and non-histone proteins is carried out by enzymes called protein arginine methyltransferases (PRMTs); this family of proteins contains nine members in mammals [2]. PRMTs facilitate the shift of a methyl group from a molecule called S-adenosylmethionine to guanidino nitrogen atoms of arginine. Methylarginines can be divided into three distinct forms depending on the number and the locations of the methyl groups: ω-N^G^-monomethylarginine (MMA), ω-N^G^,N^G^-asymmetric dimethylarginine (aDMA), and ω-N^G^,N’^G^-symmetric dimethylarginine (sDMA). PRMTs are categorized depending on their activity: Type I (PRMT1, PRMT2, PRMT3, PRMT4, PRMT6, PRMT8) catalyze MMA and aDMA forms; Type II (PRMT5, PRMT9) catalyze MMA and sDMA forms; and Type III (PRMT7) only forms MMA [3].

### 1.2. Epigenetic and Cellular Roles of PRMTs

Over 4000 proteins have been identified to be methylated on their arginine residues, thus, leading to a broad range of biological effects. PRMTs control the pre-mRNA splicing of genes involved in cell proliferation, survival, and differentiation. Arginine methylation also occurs on many ribosomal RNA-binding proteins regulating the process of protein biosynthesis. It has been shown that PRMTs participate in the control of many aspects of the DNA damage response through the methylation of key DNA repair and cell cycle checkpoint proteins: PRMT-depleted cells are hypersensitive to DNA damage. Signaling proteins can also be methylated on their arginine residues, thus, leading to a modulation of their activity, especially in different growth factor-receptor signaling pathways such as the TGFβ, the EGF, or the PDGF receptor pathways [4,5]. PRMTs are also involved in more specific cellular processes. For instance, the methylation of sirtuin 7 by PRMT6 modulates glucose metabolism and mitochondrial biogenesis. In addition, the arginine methylation of glyceraldehyde-3-phosphate dehydrogenase (GAPDH) by PRMT4 (also called coactivator-associated arginine methyltransferase 1—CARM1) has inhibitory effects on glycolysis. Actin and neuronal development are also mediated by the arginine methylation of the actin nucleator cordon–bleu WH2 repeat protein by PRMT2, thus, helping the neuronal morphogenesis [6].

**Figure 1:**
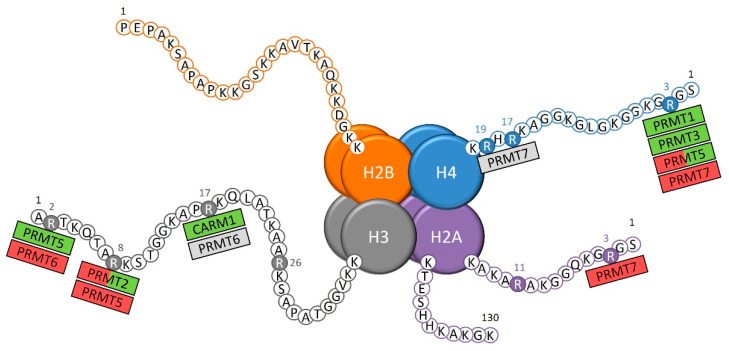
The known sites of arginine methylation on histone tails by protein arginine methyltransferases (PRMTs). Green: transcriptional activation; red: transcriptional repression; grey: unknown role on transcription.

PRMTs catalyze the methylation on histone tail arginine that is directly involved in the control of the transcription of target genes, either by compacting or decompacting the chromatin for the transcriptional repression or activation, respectively (Figure 1) [7]. Some of these histone arginine methylations contribute to an activation of the transcription of target genes, which include the arginine 3 of histone 4 asymmetrical demethylation (H4R3me2a) by PRMT1 and PRMT3 [8,9], the H3R17me2a mark catalyzed by CARM1 [10,11], and the H3R2 symmetrical demethylation (H3R2me2s) by PRMT5 [12]. The arginine methylation of histones can also be associated with a repressive chromatin state, as shown with the H4R3 and H2AR3 monomethylation by PRMT7 [13], PRMT5 H3R8me2s [14], and PRMT6 H3R2me2a marks [15].

The modification of the chromatin state through epigenetic marks on histone tails is a very active process with regular changes, thus, explaining that a same arginine methylation mark can be associated either with the activation or repression of transcription, depending on many characteristics, such as the other epigenetic marks surrounding the methylated arginine residue [16]. For instance, the H4R3me2s by PRMT5 [17,18] and the H3R8me2a by PRMT2 [19,20,21,22,23] are such epigenetic marks with dual effect. Finally, the role of certain epigenetic marks on transcription remains unclear, such as the H4R17 and H4R19 monomethylations by PRMT7 [24] and the H3R17me2a mark by PRMT6 [10].

### 1.3. PRMTs and Cancer

It has been found that expression of certain PRMT genes is altered in some cancer types such as *PRMT1* and *CARM1* overexpressed in breast and prostate cancers or *PRMT5* and *PRMT6* overexpressed in lung and blood cancers [25]. A recently published review provides an exhaustive list of the biological mechanisms in which PRMTs are involved in each cancer type [26]. In breast cancer cells, arginine methylation causes cellular proliferation and invasion through the metastasis process, thus, influencing the clinical and survival outcome of patients. These biological phenomena are the consequences of PRMTs targeting nuclear hormone receptors, tumor suppressors, splicing factors, chromatin stability proteins, and signaling pathways such as TGFβ [27]. PRMTs have also been studied in brain tumorigenesis. Glioma cells show an increased expression of *PRMT1*, and PRMT5 has been reported to be required for glial cell differentiation and disease progression. Both glioblastoma and medulloblastoma cells have increased PRMT activity through the action of PRMT5 and its association with the oncoprotein MYC [28]. Roles of PRMTs have also been characterized in pancreatic cancers, especially in pancreatic ductal adenocarcinoma (PDA) cells and tissues whereby *PRMT1* is found to be overexpressed and is associated with a shorter overall survival and a poorer differentiation status. The heat-shock protein HSP70 has been found to be methylated on specific arginine residues, leading to the regulation of PDA cell drug resistance [29].

Globally, the overexpression of PRMTs is associated with a bad prognosis, thus, explaining the interest on using inhibitors against these proteins as a novel therapeutic strategy. In this review, the roles of PRMTs in the formation of blood cells and in hematological malignancies will be discussed. Moreover, since PRMT inhibition has become a novel and promising therapeutic strategy, we will focus on PRMT inhibitors that are currently used in various models of hematological malignancies and their involvement in clinical trials.

## 2. Involvement of PRMTs in Hematopoiesis and Hematological Malignancies

### 2.1. PRMT1

PRMT1 has been identified as the predominant arginine methyltransferase in rat fibroblast cells, and it has been reported that this enzyme contributes to most activities of the PRMTs in mouse tissues [30].

In hematopoiesis, various roles of this enzyme have been demonstrated. Indeed, in hematopoietic cell differentiation, PRMT1 methylates the Runt-related transcription factor 1 (RUNX1), which is involved in hematopoiesis and myeloid differentiation. This methylation occurs on residues R206 and R210 and leads to a positive regulation of transactivation functions of RUNX1 [31]. The biological effects of R206 and R210 methylation on RUNX1 are highlighted using a new mouse model harboring a double arginine-to-lysine mutation (*Runx1^KTAMK/KTAMK^* mice). The arginine methylation of RUNX1 by PRMT1 is dispensable for T-cell development inside the thymus of these mice, although this modification seems necessary for the maintenance of the peripheral CD4^+^ T-cell population [32]. In primary human CD34^+^ cord blood cells, PRMT1-mediated RUNX1 methylation controls the transcription of the *Integrin Subunit Alpha 2b* (*CD41*) gene—an early marker of definitive hematopoiesis that is also expressed in megakaryocytic cells and multipotent progenitor cells [31]. The overexpression of PRMT1 in human CD34^+^ hematopoietic stem cells (HSCs) induces a decrease in *CD41* expression over megakaryopoiesis induced by thrombopoietin (TPO). Therefore, PRMT1 may contribute to the negative regulation of TPO-induced megakaryocytic differentiation [33]. Moreover, another recent study has shown that the pharmacological inhibition of PRMT1 enhances megakaryocytic differentiation in the context of myelodysplastic syndrome (MDS) [34].

PRMT1 expression is relatively low in long-term hematopoietic stem cells (LT-HSCs), whereas its expression is high in myeloid progenitor cells, with the highest expression found in megakaryocyte-erythrocyte progenitors (MEPs). The inhibition of PRMT1 in human CD34^+^ HSCs leads to an increase in mature megakaryocyte frequency [35]. These findings are consistent with a more recent study demonstrating that *Prmt1* knockout (KO) mice display a decrease in megakaryocyte progenitors. In addition, the depletion of *Prmt1* causes the development of strong anemia and leukopenia [36]. A chemical probe that recognizes specifically PRMT1 called E84 has been generated to define unknown hematopoietic subpopulations with unique epigenetic signatures in blood lineages. Indeed, the PRMT1 expression and staining intensity of E84 are positively correlated and Lin^−^ Sca1^+^ cKit^+^ (LSK) cells can be divided into subgroups depending on their E84 brightness. As previously shown, E84^low^ cell population contains more LT-HSCs with an enrichment of the signaling lymphocyte activation molecule family (SLAM) progenitors [37].

In more mature cell types, such as monocytes and macrophages, various functions of PRMT1 have been highlighted. PRMT1 controls major histocompatibility complex (MHC) class I through the existence of an HIF1α-PRMT1 regulatory loop and the regulation of Human leukocyte antigen (HLA)-B expression [38]. It also regulates the methylation of class II MHC transactivator (CIITA), thus, promoting its degradation [39]. In macrophages, PRMT1 methylates GAPDH, suppressing its S-nitrosylation induced by lipopolysaccharides (LPS) and interferon gamma (IFNγ), leading to the inhibition of macrophage cell death [40].

A role of PRMT1 in pre-B cells has been identified via its interaction with BTG2. The PRMT1-BTG2 complex modulates pre-B cell differentiation through the methylation of cyclin-dependent kinase 4 (CDK4), and the complex is also able to impair the pre-B acute lymphoid leukemia (ALL) induction [41]. Although PRMT1 seems dispensable for the mature B-cell number, phenotype, or distribution in the mouse spleen, this enzyme has a role in their activation and differentiation. Moreover, PRMT1 is required in B cells for several processes involved in humoral immunity [42] and for their functionality, as *Prmt1^−/−^* mice display defects in the antibody response from T-cell-independent, but not from T-cell-dependent, antigen stimulation [43].

A role for PRMT1 has also been highlighted in T cells, more precisely in T-cell polyfunctionality. Indeed, PRMT1-positive CD8^+^ T cells produce higher levels of interleukin (IL)-2 and are associated with enhanced polyfunctionality—this effect resulting from an epigenetic regulation of effector gene expression through the methylation of H4R3 by PRMT1 [44]. It has also been determined that PRMT1 could regulate Th17 differentiation by controlling the reciprocal recruitment of the signal transducers and activators of transcription STAT3 and STAT5 via H4R3 methylation, thus, resulting in the activation of *IL-17* gene expression [45]. In CD4^+^ T cells, PRMT1 methylates forkhead box P3 (FOXP3) at residues R48 and R51, and the inhibition of their interaction leads to a decrease in CD4^+^ T-cell activity, showing that the arginine methylation of FOXP3 accentuates suppressive functions of T cells [46]. GFI1 is a transcriptional factor expressed in T cells and has a role in the regulation of cellular processes such as DNA damage signaling and repair protein expression. The requirement of GFI1 for the binding of PRMT1 with its substrates MRE11 and 53BP1 allows their methylation [47].

In addition to normal hematopoiesis, PRMT1 has a central position in leukemogenesis, and its roles have been characterized in acute myeloid leukemia (AML) and ALL.

In mixed-lineage leukemia (MLL)-rearranged ALL, the methylation of the tyrosine kinase FLT3 on arginine R972 and R973 by PRMT1 controls leukemia cell maintenance in vitro. FLT3-arginine-mutated ALL cell transplantation significantly extended mouse survival compared to FLT3 wild-type (WT) cells. *PRMT1* knockdown favors the eradication of MLL-rearranged ALL cells as well as its inhibition, especially when combined with a tyrosine kinase inhibitor [36]. In the context of AML, PRMT1 also catalyzes the methylation of the FLT3-ITD^+^ fusion protein at residues R972 and R973. *PRMT1* knockdown blocks leukemic cell survival and growth with a more potent inhibitory effect in FLT3-ITD^+^ cells than in FLT3 WT AML cells [48]. In LSK-derived MLL-cancer stem cells, the PRMT1 function is coregulated by β-catenin and Hoxa9; by mediating similar signaling pathways as β-catenin and Hoxa9, PRMT1 is directly involved in leukemic cell self-renewal [49].

In AML, the MLL-ELN fusion protein cooperates with PRMT1 to promote H4R3 methylation as an oncogenic transcriptional regulatory complex. The direct fusion of MLL with PRMT1 increases hematopoietic cell self-renewal [50]. KMT2A-GAS7 (MLL-GAS7) or KAT6A-NCOA2 (MOZ-TIF2) AML mouse models reveal that the recruitment of PRMT1 is necessary but not sufficient for leukemia induction. The co-recruitment of PRMT1 with the histone demethylase KDM4C by the AML fusion proteins controls the H3K9me3 status of target genes. The suppression of *PRMT1* inhibits leukemogenesis and increases cell apoptosis in these two AML fusion protein models [51].

RUNX1-RUNX1T1 (RUNX1-ETO) is another fusion protein resulting from the t(8;21)(q22;q22) translocation in AML. AE9a is a splice isoform of RUNX1-ETO and is methylated by PRMT1 at residue R142, thus, activating the transcription of AE9a target genes. Knockdown of *PRMT1* suppresses proliferation and decreases the self-renewal of progenitor cells [52].

RNA splicing represents another process regulated by PRMT1 through the methylation of the RNA-binding protein RBM15 at R578 in acute megakaryocytic leukemia. PRMT1 physically interacts with the E3 ligase responsible for RBM15 ubiquitination leading to its degradation, thus, inhibiting the megakaryocytic differentiation [35]. This PRMT1/RBM15 axis for megakaryopoiesis regulation was also later described in normal human HSCs [53]. It has also been demonstrated that PRMT1 is involved in RNA splicing in ALL, and elevated levels of *PRMT1*, *serine and arginine-rich splicing factor 1* (*SRSF1*) mRNA are detected in newly diagnosed pediatric ALL samples compared to complete remission samples. Interestingly, the two proteins interact in ALL in vitro models. SRSF1 has an anti-apoptotic role and could contribute to leukemogenesis by cooperating with PRMT1 [54].

### 2.2. CARM1 (PRMT4)

CARM1 is essential for the survival of early T-cell progenitors but is dispensable for their differentiation. CARM1 modulates hematopoietic progenitor cell activity and cellularity in the fetal liver and in the bone marrow. In *Carm1* KO mice, thymopoiesis is blocked between the DN1 and DN2 stages [55] as the thymocyte cyclic AMP-regulated phosphoprotein is methylated by CARM1 at residue R650 in immature T cells. This methylation leads to an increase in early thymocyte progenitor differentiation. Moreover, *Carm1* KO mice bear aberrant T-cell development, underlining the importance of CARM1 in the promotion of thymocyte differentiation [56].

*CARM1* gene expression increases after the CD3/CD28 activation of T cells. As a result, in lymphoma cell lines stimulated with LPS, CARM1 expression is augmented, triggering caspase 3 activation, resulting in lymphocyte cell death. Similar results are obtained in spleens of mice injured either with LPS or in a polymicrobial sepsis model [57]. The activation of MHC II through CIITA-dependent transcription is enhanced by the methyltransferase activity of CARM1, as also demonstrated for PRMT1 [39]. CIITA-dependent transcription is mediated by the methylation of the CREB-binding protein by CARM1 at residues R714, R742, and R768—this methylation is required for its association with the HLA-DRA promoter [58].

CARM1 is also important in erythropoiesis through its direct interaction with the chromatin remodeler Mi2. CARM1 and Mi2 form a complex with the transcription factor c-Myb and regulate the c-Myb target gene’s transcription in several leukemia cell lines. The depletion of either CARM1 or Mi2 in erythroleukemia cell lines results in proliferation and differentiation deregulation, as seen with the depletion of c-Myb in the same models [59]. AML initiation is dependent on CARM1. Indeed, *Carm1*-deficient mice failed to develop leukemia even one year after transplantation. It is also essential for AE9a-driven leukemia maintenance, as mice depleted for Carm1 show significant improvements in survival compared to wild types. This effect is due to the methyltransferase activity of CARM1 on fusion oncoproteins [60].

PRMTs can be post-transcriptionally regulated by microRNAs (miRNAs), modulating their methyltransferase activity. CARM1 can repress miR-223 expression through the methylation of RUNX1 at residue R223, leading to a blockade of myeloid differentiation, and conversely, CARM1 expression is repressed post-transcriptionally by miR-223. The knockdown of *CARM1* induces myeloid differentiation of AML cell lines and reduces the leukemia burden in vivo [61].

### 2.3. PRMT5

**Figure 2:**
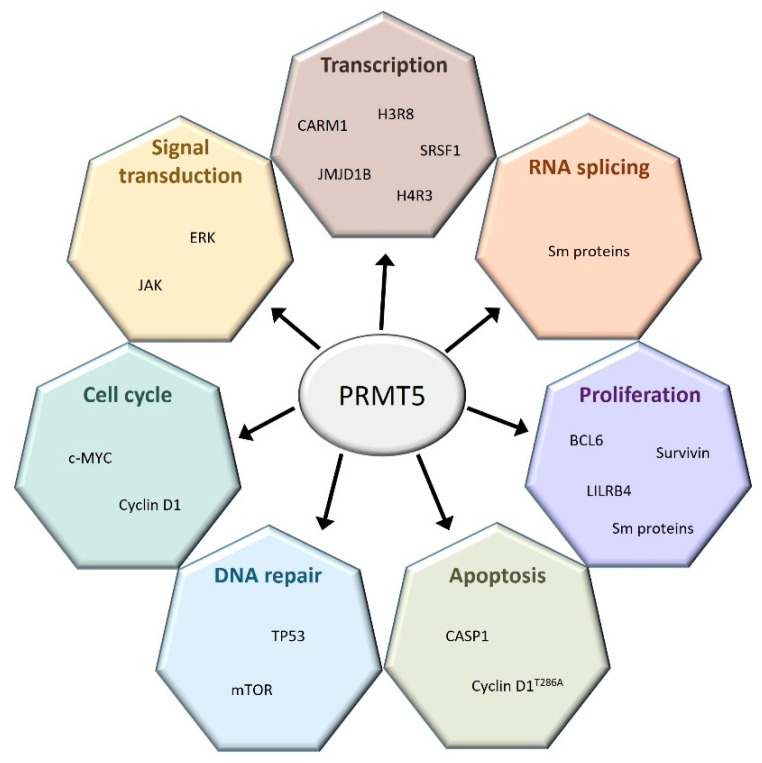
PRMT5 regulates a broad spectrum of cellular processes. PRMT5 interacts with many coding and non-coding genes involved in a large set of cellular functions.

PRMT1 and PRMT5 are the most studied PRMTs among the nine members of this protein family. The precise roles of PRMT5 are well known both in hematopoiesis and in several hematological malignancies (Figure 2).

In normal hematopoietic cells, PRMT5 is responsible for the negative regulation of HSCs and progenitor cell proliferation as its overexpression leads to a decreased colony formation ability of the cells. Alterations of both erythroid and granulomonocytic differentiations when *PRMT5* is knocked down are also reported. The phosphorylation of PRMT5 by the Janus kinase 2 active mutant JAK2^V617F^ impairs its methylation activity, resulting in the control of hematopoietic cell populations and differentiation pathways [62]. It was further demonstrated that deletion of *Prmt5* in mice provokes severe pancytopenia and loss of hematopoietic progenitor cells to the benefit of LSK and LT-HSCs. The loss of Prmt5 deeply affects erythroid differentiation in bone marrow and early thymocyte development in the thymus. In bone marrow cells, Prmt5 is pivotal to maintain cytokine signaling as deletion of this methyltransferase produces a decrease in the expression of several cytokine receptors [63]. Furthermore, Prmt5 depletion in mice impairs the genomic stability of hematopoietic stem and progenitor cells (HSPCs), inducing increased DNA damage accumulation and HSPC cell cycle arrest. This effect is due to an overactivation of the p53 pathway, an activation of mTOR signaling as well as a splicing defect of key DNA repair factors, as demonstrated for the lysine acetyltransferase TIP60/KAT5 [64,65]. The PRMT5 methyltransferase can cooperate with the lysine demethylase JMJD1B to control the symmetric demethylation of H4R3 at promoters of hematopoietic genes, provoking a complex transcription regulation of key genes for HSPC survival, proliferation, and differentiation [66].

Zebrafish models have shown that *prmt5* is necessary for blood cell formation, although it is dispensable for blood vessel formation. Prmt5 promotes vascular morphogenesis through the regulation of gene expression by acting as a scaffold protein for chromatin structure [67].

PRMT5 not only has a role in HSPCs but also in T-cell differentiation and more precisely in the CD8^+^ T-cell development. The T-cell-specific depletion of PRMT5 enforces differentiation into Klrg1^+^ effector T cells through the loss of the epigenetic marks H4R3me2s and H3R8me2s, enhancing *B lymphocyte-induced maturation protein-1* transcription [68].

In multiple myeloma (MM) patients, PRMT5 is often overexpressed and is associated with decreased progression-free survival and overall survival [69]. In MM cell lines, PRMT5 controls pyroptosis through the silencing of caspase 1 (*CASP1*). The knockdown of *PRMT5* induces CASP1 and cleaved CASP1 expression, and pyroptosis could be rescued in this model by knocking down *CASP1* [70].

In B-cell chronic lymphoid leukemia (CLL) cell lines, the overexpression of *PRMT5* is due to its enhanced translation, caused by an alteration of the expression of miR-19a, -25, -32, -92b, and -96, which targets PRMT5. The upregulation of its expression leads to the silencing of PRMT5 target genes through its epigenetic role at H4R3 and H3R8, especially the retinoblastoma family of tumor suppressor genes [71].

Similar observations reported in mantle cell lymphoma (MCL) show that MCL cell lines with an enhanced PRMT5 expression display aberrant expressions of miR-92b and miR-96. In MCL patient samples, increased PRMT5 expression inhibits the *suppressor of tumorigenicity 7* (*ST7)* gene expression via an altered H4R3 and H3R8 methylation profile. Interestingly, the knockdown of *PRMT5* impairs transformed B-cell proliferation and growth [72]. It was further discovered that knockdown of *PRMT5* in B-cell lymphoma cell lines epigenetically increases the expression of miR-33b and miR-96 as previously described, and miR-503. These miRNAs are predicted to bind both Cyclin D1 and c-MYC to reduce their expressions, thus, decreasing B-cell lymphoma cell survival [73].

PRMT5, a driver oncogene in cooperation with nuclear Cyclin D1T286A is also involved in other driver oncogene pathogenesis in aggressive T-cell lymphoma and leukemia. Cyclin D1^T286A^ activates PRMT5 through the phosphorylation of the methylosome protein 50 (MEP50), leading to methylation followed by an inhibition of p53-dependent apoptosis of leukemia and lymphoma cell lines [74].

In non-Hodgkin lymphoma (NHL), PRMT5 modulates the TP53/NF-κB p52/BCL3 pathway, which enhances Cyclin D1 proliferative signaling. In addition, the overexpression of PRMT5 epigenetically reduces retinoblastoma proteins’ RB1 and RBL2 activity and increases the Polycomb-repressive complex 2 (PRC2) expression in NHL patient samples [75]. This enhanced Cyclin D1, c-MYC, and Survivin expression results from an epigenetic repressive role of PRMT5 at WNT/β-Catenin antagonist *AXIN2* and *WIF1* promoters. This repression generates a constitutive activation of AKT/GSK3 and WNT/β-Catenin signaling pathways [76].

During lymphomagenesis, MYC directly upregulates PRMT5 expression, thus, protecting Sm protein activity, leading to the transcription of genes involved in RNA splicing, cell survival, and proliferation. Anti-proliferative and anti-apoptotic effects are observed in *PRMT5*-depleted B-cell lymphoma using antisense oligonucleotides through the alteration of splicing of genes involved in lymphomagenesis [77]. Following a B-cell receptor activation and triggering of the PI3K/AKT signaling pathway, MYC-mediated *PRMT5* transcription induces cell cycle progression, increasing cell survival and proliferation in both activated B-cell (ABC) and germinal center (GC) diffuse large B-cell lymphoma (DLBCL). In ABC DLBCL, *PRMT5* transcription is also stimulated through the activation of the BTK-mediated NF-κB pathway [78]. In germinal centers, B-cell lymphoma 6 (BCL6) is methylated at residue R305 by PRMT5, giving rise to GC formation, affinity maturation, and B-cell lymphoma survival and proliferation [79].

In leukemia cell lines, the H3R2 and H3R8 symmetrical dimethylation by PRMT5 antagonizes H3K27 trimethylation by PRC2, thus, maintaining the transcription of target genes, resulting in proliferative effects and cell cycle activation [80].

PRMTs can also interact with each other as it was recently discovered for PRMT5. Indeed, PRMT5 methylation at residue R505 by CARM1 in vitro is essential for its methyltransferase activity, especially on histone 4 at the promoters controlling the β-globin gene (*HBB*) expression, thus, reducing the latter [81].

Various roles of PRMT5 have also been recently characterized in AML, and its methyltransferase activity is important for the development of the MLL-AF9 rearranged types of AML. New substrates have been identified, including many proteins involved in mRNA end processing, splicing, and binding. In AML cell lines, the methylation of SRSF1 by PRMT5 affects its binding to mRNAs and proteins [82]. In MLL-AF9 AMLs, the PRMT5 expression depends on CDC73, which is part of the PAF complex to promote the expression of other oncogenic factors such as *STAT5* and *HOXA9* [83]. The overexpression of PRMT5 leads to an increase in leukemia cell growth, especially in FLT3-ITD primary blasts compared to FLT3-WT patients. Conversely, the depletion of PRMT5 significantly decreases AML cell growth either in vitro or in vivo [18]. Patients with newly diagnosed or recurrent AML display an increased expression of *PRMT5*, and a positive correlation is found with the expression of the *leukocyte immunoglobulin-like receptor B4* (*LILRB4*). *LILRB4* expression is triggered by the activation of the mTOR pathway by PRMT5, enhancing the invasion of AML cells [84].

PRMT5 is also highly expressed in acute promyelocytic leukemia (APL), an AML subtype mainly driven by the PML-RARα oncoprotein. PRMT5 suppresses both the ubiquitination and degradation of PML-RARα and inhibits its interaction with the E3 ubiquitin ligase RNF4 through its methylation, thus, contributing to leukemic cell proliferation [85].

### 2.4. PRMT6

Little is known about the role of PRMT6 in hematopoiesis or hematological malignancies. In AML, demethylated H3R2 is considered as the main methylation site of PRMT6 in vivo. The activity of this enzyme is reduced in the presence of the H3K4 or H3K9 methylation marks, but can be slightly enhanced when H3K27 is methylated [86]. The inhibition of H3R2me2a by H3K4 trimethylation is the consequence of the binding of an MLL methyltransferase complex involving WDR5. Conversely, PRMT6 inhibits the transcription of genes known to be regulated by H3K4me3 such as Hox- and Myc-dependent genes by preventing the interaction of MLL complexes with the histone 3 tail [4].

The knockdown of *PRMT6* in erythroleukemic cells resulted in a significantly decreased proliferation and an increase in cells in the G1 phase of the cell cycle due to a reduced *Cyclin D1* expression. In normal leukemic cells, LEF1 (lymphoid enhancer-binding factor-1) interacts with PRMT6, provoking its recruitment at the *Cyclin D1* promoter [87].

PRMT6 is also able, like PRMT1 [31], to interact with RUNX1, negatively affecting megakaryocytic gene expression, thus, repressing the differentiation of human CD34^+^ progenitor cells. Upon megakaryocytic differentiation by TPO, the H3R2 methylation mark is lost for the benefit of the H3K4 methylation increasing the *inter alia IL6ST* gene expression through the action of the protein arginine deiminase PADI4 at *SCL/TAL1 (stem cell leukemia/T-ALL-1*)-target gene expression activation [88,89]. More recent data confirmed that PRMT6 has an important role in the regulation of erythroid differentiation in primary human CD34^+^ cells via its recruitment as a repressor at the promoters of erythroid genes such as *glycophorin A* (*GPA*), whose expression is controlled by SCL/TAL1 [90]. There is an increased RUNX1 and PRMT6 co-occupancy at the *KLF1* promoter upon megakaryocytic differentiation—KLF1 is a transcription factor also involved in the maturation of erythroid cells. The inhibition of PRMT6 could promote erythroid differentiation by decreasing the repressive function of the RUNX1 and PRMT6 concomitant actions at the *KLF1* promoter [91].

### 2.5. PRMT7

PRMT7 is the only known PRMT with a single monomethylation activity on arginine. Activation of the NF-κB pathway in monocytes leads to *PRMT7* transcription, thus, increasing the monomethylation of histones. Moreover, a reduced expression of *PRMT7* is correlated with a decreased recruitment of monocytes at the injury site, suggesting that PRMT7 could promote monocyte migration [92].

Conditional *CD19* KO mice for Prmt7 (*Prmt7*-CKO) display normal B-cell differentiation in bone marrow and the impairment of mature B-cell formation in the spleen. Moreover, the loss of PRMT7 promotes GC formation and, as shown for PRMT5, PRMT7 can bind to the promoter of *Bcl6*, the master regulator of GC maintenance, to inhibit its functions, leading to a regulation of GC formation [93].

High levels of *PRMT7* transcripts are found in T-ALL, especially in mature subtypes of T-ALL. The genetic depletion of *Prmt7* reduces colony formation and cell viability of T-ALL cells, and most of the differentially monomethylated proteins in *Prmt7* KO cells belong to protein complexes that affect RNA and DNA processing. More importantly, these cells harbor an altered RUNX1 monomethylation status, thus, deregulating the RUNX1-target gene expression. Among them, *BCL11A* and *DNTT,* which are regulators of T-cell development, are identified, indicating that PRMT7 has an indirect role in the pathogenesis of T-ALL through RUNX1 monomethylation [94].

Targeting *PRMT7* through genetic deletion or inhibition delays chronic myeloid leukemia (CML) development and prevents the self-renewal of leukemic stem cells (LSCs) both in mouse and human CML models. The loss of PRMT7 functions results in reprogramming glycine metabolism that induces the generation of toxic methylglyoxal, hence, the eradication of LSCs [95].

### 2.6. Other PRMTs

The analysis of RNA sequencing data from *RUNX1*-mutated AML patients revealed that *PRMT8* is found among the top 10 of *RUNX1* mutation-associated hub genes [96]. *PRMT8* and *PRMT3* have also been identified as downregulated tumor suppressor genes in pediatric acute monoblastic leukemias [97]. Furthermore, a real-time PCR array has shown that *PRMT2* seems to be downregulated in pediatric ALL [98].

## 3. Pharmacological Inhibition of PRMTs in Hematological Malignancies

Table 1 presents the chemical tools that have been developed to inhibit the activity of PRMTs in hematological malignancies. They have been tested in several models including primary samples from patients, xenografts of primary specimens into immunodeficient mice, mouse models of hematological malignancies, and human cell lines.

Several PRMT1 and Type I inhibitors exhibit the same effects on AML cell lines with an inhibition of cell growth and a decreased viability of cells [48,51,52,100,101,105]. In mouse AML xenografts, an increase in the overall survival of mice is demonstrated [51]. This effect is synergistic when combined with a tyrosine kinase inhibitor (TKI) in patient-derived xenograft (PDX) models of AML [48] and ALL [36].

Several CARM1-specific inhibitors have also been developed. They show decreased growth of NHL [108], MM [107], and viability of AML cell lines [60]. The gavage of mice with the orally bioavailable CARM1 inhibitor EZM2302 improves survival in an AML model [60] and decreases growth in NHL [108] and MM mouse models [106]. Involvement of CARM1 in the control of the cell cycle is also highlighted, as inhibited cell lines reveal an increased cell cycle arrest in MM [107] and NHL models [108].

In the past few years, PRMT5 inhibitors have been widely tested, especially on AML and NHL. A decrease in cell viability and cell proliferation as well as an increase in apoptosis and cell cycle arrest have been demonstrated in both AML and NHL cell lines treated with PRMT5 inhibitors [18,76,79,80,82,83,110,113,114,119]. PRMT5 inhibition in AML cell lines also leads to an increase in DNA damage in the cells, thus, explaining the increase in leukemic cell death. Indeed, PRMT5 inhibition can sensitize leukemic cells to PARP inhibitors while sparing the normal hematopoietic compartment [64]. As previously described in AML, treatment with a PRMT5 inhibitor impairs DNA repair mechanisms in CLL cell lines. This effect is enhanced when combined with a PARP inhibitor, leading to increased cell death [111].

In MM cell lines, treatment with a PRMT5 inhibitor decreases the levels of H4R3me2s at the *CASP1* promoter, thus, enhancing the pyroptosis of myeloma cells [70]. A decrease in cell proliferation and an increase in apoptosis are also demonstrated in MM with a PRMT5 inhibitor. Moreover, orally treating mice reduces MM growth in vivo [69].

A decrease in a colony-forming ability was observed in CML cell lines and human primary CML cells treated with a PRMT5 inhibitor [115]. PRMT5 inhibition has also been studied in mouse models. A prolonged overall survival of treated mice associated with fewer LSCs is shown in CML—this effect is enhanced in combination with a TKI [115]. Inhibiting PRMT5 in mouse models of NHL and AML extend the survival of mice and promote apoptosis [78,83,113].

PRMT5 inhibition has also been studied in myeloproliferative neoplasms (MPN), especially in JAK2-mutated MPN with a decreased proliferation both in vitro and in patient samples. There is an altered methylation of the E2F1 transcription factor, thus, impacting the cell cycle and DNA damage repair target genes. When combined with JAK1/2 inhibitors, PRMT5 inhibition significantly increases the cell death of MPN cells in vivo [118].

So far, only one PRMT7 inhibitor has been tested in different CML models. JS1310 treatment significantly increases the survival of CML mice and reduces splenomegaly, tumor burden, as well as the number of leukemic stem and progenitor cells in bone marrow and the spleen. Primary CML CD34^+^ cells display increased apoptosis and a lower colony-forming ability when treated with the PRMT7 inhibitor. When xenografted into mice, JS1310-treated primary CML cells induce a decrease in myeloid cell formation [95].

Other PRMT inhibitors have been developed, but studies on their effects on hematological malignancies are restrained. In this context, PRMT1 inhibitors E-84 [120], K313 [121]; the CARM1 inhibitor compound 49 [122]; and the PRMT5 inhibitors Y2431 [123], C_4 [119], JNJ-64619178 [124], compound 5, and compound 19 [125] should be mentioned. In addition, we must mention that the development of the transition state mimics potent CARM1 inhibitors from PABP1-derived peptides that are covalently linked to an adenosine moiety [126].

## 4. Clinical Trials

Among all the anti-PRMT molecules developed so far, PRMT5 inhibitors represent the most promising potential therapeutic drugs. PRMT5 is the most-expressed PRMT in a large set of cell types and human tissues, and the various roles of PRMT5 are well characterized either in normal hematopoiesis or in hematological malignancies. Moreover, Type I PRMT and specific PRMT5 inhibitors have shown strong results both on leukemia and lymphoma cell lines and in several mouse models of hematological malignancies, thus, explaining their use in clinical trials. There are currently six clinical trials involving Type I and PRMT5 inhibitors in hematological malignancies (Table 2).

Five out of the six trials are ongoing, with one study (NCT03666988) that is terminated and one study (NCT02783300) that has preliminary results presented in *Annals of Oncology* [127]. These two studies report that even if the use of PRMT inhibitors on patients show promising results for the treatment of hematological malignancies, most patients experienced at least one treatment-related adverse event (TRAE). The most common TRAEs were grade 1 or 2 (including fatigue, anemia, nausea, alopecia) or grade 3 or 4 (including anemia, thrombocytopenia, neutropenia, fatigue). There were no grade 5 related TRAE in either of these studies. Overall, investigators highlight that adverse events are common but manageable.

## 5. Conclusions

**Figure 3:**
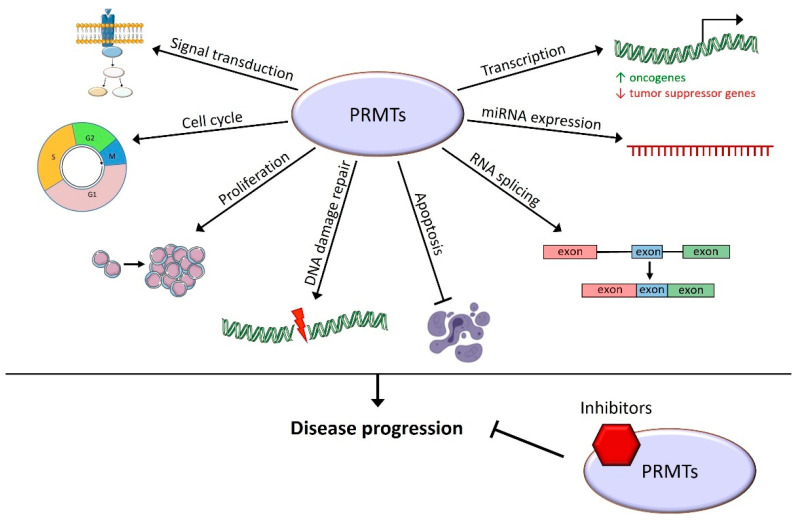
Cellular processes known to be controlled by arginine methylation in hematological malignancies. PRMTs play roles in many cellular functions leading to hematological malignancy progression, and their inhibition has a promising therapeutic effect.

This review summarizes the roles of PRMTs in hematopoiesis and in the different existing hematological malignancies. The importance of arginine methylation for the regulation of HSC differentiation, proliferation, and self-renewal has been highlighted. In a various set of hematological malignancies, they allow the disease progression through the methylation of histone tails that control transcription activation or the repression of target genes. They are also able to directly methylate transcription factors or proteins involved in various cellular processes: RNA splicing and end-processing, DNA damage repair processes, proliferation, cell cycle, and signal transduction in various signaling pathways (Figure 3).

Recently, specific pharmacological PRMT inhibitors have been developed. They participate in a significant improvement in patient conditions in many solid cancers, especially PRMT5 inhibitors that are currently involved in many phase I or II clinical trials. Inhibitors directed against PRMTs type I, i.e., PRMT1, CARM1, PRMT5, and PRMT7, have been tested on hematological malignancy models and have shown a significant decrease in malignant cell proliferation and an increased apoptosis. In mouse models of hematological malignancies, PRMT inhibition leads to an increase in overall survival. There are also ongoing clinical trials involving PRMT5 inhibitors in hematological malignancies, showing, once again, that PRMT inhibition might become a useful therapeutic tool for cancer treatment.

## Figures and Tables

**Table 1:** Tool compounds for PRMT inhibition in hematological malignancies.

PRMT(s) Inhibited	Drug (Alias)	IC_50_	Disease	Experimental Models	References
Type I PRMTs	MS023 [99]	30 nM (PRMT1) 119 nM (PRMT3) 83 nM (CARM1) 4 nM (PRMT6) 5 nM (PRMT8)	ALL	Cell lines; primary specimens; PDX	[36]
			AML	Cell lines; primary specimens; PDX	[48,100]
	Compound 28d [101]	1.1 nM (PRMT1) 22 nM (PRMT3) 63 nM (CARM1) 1.2 nM (PRMT6) 1.1 nM (PRMT8)	MLL	Cell lines	[101]
PRMT1	AMI-408 (compound 4) [102]	ND	AML	Cell lines; mice xenografts	[51]
	TC-E 5003(compound 2e) [103]	1 µM	ALL	Cell lines	[36]
			AML	Cell lines	[48]
	RM-65 [104]	55 µM	AML	Cell lines	[52]
	DB75 (Furamidine) [105]	9.4 µM	AMKL	Cell lines	[35]
CARM1	EPZ025654 (GSK35336023) [106]	3 nM	AML	Cell lines; primary specimens; PDX	[60]
	TP-064 [107]	<10 nM	NHL	Cell lines	[108]
			MM	Cell lines	[107]
	EZM2302 (GSK3359088) [106]	6 nM	MM	Cell lines; xenografted mice gavage	[106]
			AML	Transplanted mice gavage	[60]
			NHL	Transplanted mice gavage	[108]
PRMT5	EPZ015666 (GSK3235025) [109]	22 nM	AML	Cell lines; primary specimens; transplanted mice gavage	[82,83,100,110]
			CLL	Cell lines	[111]
			NHL	Cell lines; PDX	[78]
			MM	Cell lines; primary specimens; xenografted mice gavage	[69]
	EPZ015866 (GSK3203591) [112]	4 nM	NHL	Cell lines; primary specimens	[79,113]
			MM	Cell lines	[70]
			AML	Cell lines	[100,114]
	EPZ015938 (GSK3326595) [113]	6.2 nM	NHL	Cell lines; PDX; transplanted or xenografted mice gavage	[78,113]
	GSK3186000A (compound 14) [112]	11 nM	AML	Cell lines	[64,80]
	PJ-68 [115]	517 nM	CML	Cell lines; primary specimens; intraperitoneally injected transplanted mice	[115]
	HLCL-61 [18]	7.21–21.46 µM	AML	Cell lines; primary specimens	[18]
	LLY-283 [116]	22 nM	AML	Cell lines	[114]
	CMP-5 [117]	ND	NHL	Cell lines; mouse primary specimens	[76]
	C220 [118]	ND	MPN	Cell lines; transplanted mice gavage	[118]
PRMT7	JS1310 [95]	5 µM	CML	Leukemic mice; primary specimens; PDX	[95]

ALL: acute lymphoid leukemia; AML: acute myeloid leukemia; MLL: mixed lineage leukemia; ND: not determined; PDX: patient-derived xenograft; AMKL: acute megakaryoblastic leukemia; NHL: non-Hodgkin lymphoma; MM: multiple myeloma; CLL: chronic lymphoid leukemia; CML: chronic myeloid leukemia; MPN: myeloproliferative neoplasm.

**Table 2:** Clinical trials involving PRMT inhibitors in hematological malignancies.

Trial Number	Official Title of the Study	Number of Patients	Drug (Alias)	PRMT Inhibited	HM Included
NCT03666988	A Phase I, open-label, dose-escalation study to investigate the safety, pharmacokinetics, pharmacodynamics, and clinical activity of GSK3368715 in patients with solid tumors and DLBCL.	31	GSK3368715 (EPZ019997)	Type I PRMTs	DLBCL
NCT03614728	A Phase I/II Study to investigate the safety and clinical activity of GSK3326595 and other agents in patients with MDS and AML.	30	GSK3326595 (Pemrametostat)	PRMT5	MDS; CMML; AML
NCT02783300	A Phase I, open-label, dose-escalation study to investigate the safety, pharmacokinetics, pharmacodynamics, and clinical activity of GSK3326595 in patients with solid tumors and NHL.	322	GSK3326595 (Pemrametostat)	PRMT5	NHL
NCT03573310	A Phase I, first in-human, open-label study of the safety, pharmacokinetics, and pharmacodynamics of JNJ-64619178, an inhibitor of PRMT5 in patients with advanced cancers.	114	JNJ-64619178 (Onametostat)	PRMT5	B NHL; lower risk MDS
NCT03886831	A Phase I, open-label, multicenter, dose-escalation, dose-expansion study of PRT543 in patients with advanced solid tumors and hematologic malignancies.	227	PRT543	PRMT5	DLBCL; myelodysplasia; myelofibrosis; MCL; AML; CML
NCT04089449	A Phase I, open-label, multicenter, dose-escalation and -expansion study of PRT811 in subjects with advanced solid tumors, CNS lymphoma, and recurrent high-grade gliomas.	145	PRT811	PRMT5	CNS lymphoma

HM: hematological malignancies; DLBCL: diffuse large B-cell lymphoma; MDS: myelodysplastic syndrome; CMML: chronic myelomonocytic leukemia.
